# Association between housework and the risk of dementia among older Chinese adults: a prospective cohort study

**DOI:** 10.3389/fpsyg.2023.1228059

**Published:** 2023-07-24

**Authors:** Yuanlong Wang, Xinxin Luo, Xiangyun Long, Yuan Shao, Song Zhang, Yingli Zhang, Yongjun Wang

**Affiliations:** ^1^School of Mental Health and Psychological Sciences, Anhui Medical University, Hefei, China; ^2^Shenzhen Mental Health Centre, Shenzhen Kangning Hospital, Shenzhen, China; ^3^Anning Mental Rehabilitation Hospital, Changchun, China

**Keywords:** Dementia, Aging, housework, cognitive impairment, older Chinese adults, psychology

## Abstract

**Objectives:**

Physical activity (PA) is known to improve physical functioning and mental health and to reduce the incidence of dementia. However, studies of the effects of non-recreational PA on the incidence of dementia, especially in East Asian populations, remain limited. In this study, we evaluate the association of doing housework with the risk of dementia among participants in the Chinese Longitudinal Healthy Longevity Survey (CLHLS).

**Methods:**

The analysis was conducted with data from 7,237 CLHLS participants age over 65 obtained in 2008/2009, 2011/2012, 2014, and 2018. The frequency of housework performance was classified into four groups. A Cox proportional-hazards model was used to examine the association of the baseline housework frequency with the incidence of dementia, with adjustment for demographic and socioeconomic characteristics and lifestyle and health conditions.

**Results:**

The adjusted multivariate model showed that the incidence of dementia was lower among participants who did housework almost every day than among those who rarely or never did housework (hazard ratio = 0.49; 95% confidence interval, 0.39–0.61). The subgroup and sensitivity analyses yielded similar results.

**Conclusion:**

A high frequency of housework performance was associated with a reduced incidence of dementia among older Chinese adults, especially those who did not exercise regularly. The encouragement of engagement in housework would be a cost-effective measure promoting healthy aging in the Chinese population.

## Introduction

Dementia is a common neurodegenerative disease that mainly presents as a slow and continuous cognitive decline, especially memory loss, varying degrees of personality change, and declining ability to perform activities of daily living (ADL) (Tanaka et al., 2020; Gonzales et al., 2022), with the aging of the global population, the World Alzheimer Report predicts that the number of people with dementia worldwide will increase from about 50 million in 2018 to 82 million by 2030 and surpass 152 million by 2050 (Alzheimer’s Disease International, 2018). China is the country with the largest number of patients with dementia; its population aged > 60 years includes about 15.07 million of these patients (Jia et al., 2020). The provision of care and support to people with dementia places huge burdens on families, health care systems, and society at large (Etters et al., 2008; Livingston et al., 2017). A previous cohort study of 431,924 participants showed that sustained physical activity was associated with a reduced risk of developing dementia (Huang et al., 2022). Thus, it is particularly important to research dementia risk factors and prevention methods.

Physical activity (PA) is of great benefit to our health, about 40% of dementia cases worldwide can be attributed to 12 modifiable risk factors. Exercise, and more broadly (PA), plays a significant role (Livingston et al., 2020). For older adults, PA is critical to the relationship among disease prevention, the maintenance of independence, and the improvement of the quality of life (Northey et al., 2018; Bangsbo et al., 2019). Regular PA can effectively prevent falls, chronic diseases, and death in this population (Kyu et al., 2016; Ng et al., 2020; Sherrington et al., 2020; Stensvold et al., 2020); from a psychological perspective, it can effectively prevent cognitive impairment and improve mental health (Herring et al., 2012; Northey et al., 2018; Bangsbo et al., 2019), this may be with that PA effectively reduces peripheral concentrations of pro-inflammatory cytokines and improves peripheral brain-derived neurotrophic factor (BDNF) levels, exerting neuroprotective effects (Nascimento et al., 2014; Loprinzi and Frith, 2019). PA also affects other factors related to cognitive function, including the cholesterol, testosterone, estradiol, and insulin levels (Jensen et al., 2015).

Physical activity also reduces the risk of dementia, earlier researchers found that heavy housework (three or more times per week) was associated with a 21% reduction in dementia risk (Bowen, 2012). Subsequent researchers used data from 2005 to collect MMSE scores for 454 volunteers who agreed to undergo a clinical evaluation and brain donation and collected daily activity counts by an omnidirectional accelerometer worn on the nondominant wrist, performed brain removal, tissue sectioning, and preservation after the volunteers died, found that higher daily activity level was independently associated with a higher cognitive level. However, the association of the summary measure of AD pathology and cognitive function did not vary with the level of physical activity (Buchman et al., 2019). Those findings provide a new perspective for subsequent researchers to explore dementia prevention beyond hormonal therapy and anti-inflammatory drugs. However, although both housework and PA are a physical exercise, but there are some differences between housework and PA, researchers have not yet determined whether housework does the same role as PA, but these types of activities such as sweeping and washing provide a certain degree of PA, reducing sedentary behavior and time (Lawlor et al., 2002), now studies of housework and the incidence of dementia remain limited, especially in China, and those from other countries have yielded inconsistent results (Hank and Jürges, 2007; Gimenez-Nadal and Molina, 2015; Lee et al., 2021; Zhu et al., 2022). The heterogeneous findings may be related to the cross-sectional nature of most existing studies. In addition, the frequency of housework has not been quantified, samples have been heterogeneous and small, and representativeness has been insufficient due to cultural influences on housework distribution and activities. Thus, more longitudinal and interventional studies are necessary for examining the association between housework and older adults’ health.

In this study, we applied the Chinese Longitudinal Healthy Longevity Survey (CLHLS) to explore the relationship between housework frequency and dementia risk (Gu et al., 2017). We explored this risk for people who did housework at different frequencies with reference to those who rarely or never did housework, and examined whether age, gender, and regular exercise impacts this association. Finally, we performed a sensitivity analysis of the reverse causal effect and post-stroke dementia.

## Methods

### Study population

The CLHLS was conducted in China by the Development Research Center/National Development Research and Peking University from 1998 to 2018. People in about half of the counties and cities in 23 provinces were selected randomly to participate in eight surveys over time. The CLHLS is the earliest and longest-running survey in China aimed at understanding the basic personal and family conditions, socioeconomic backgrounds, health-related quality of life, and longevity of older adults to promote healthy aging (Zeng, 2012). Similar research approaches have been described elsewhere (Deng et al., 2020). The information recruited by the respondents was structured questionnaire data from a face-to-face survey by trained professionals. When the respondents could not answer the questions, they were usually answered by their family members and spouses, but the questions of cognitive function and emotion must be answered by the respondents themselves, and the resulting data have been found to be of good quality (Lv et al., 2018). The ADL measure in the CLHLS was based on dressing, bathing, indoor transfer, eating, continence, and toilet use; participants unable to complete all of these tasks independently were considered to have an ADL disability (Katz et al., 1963).

**Figure 1:**
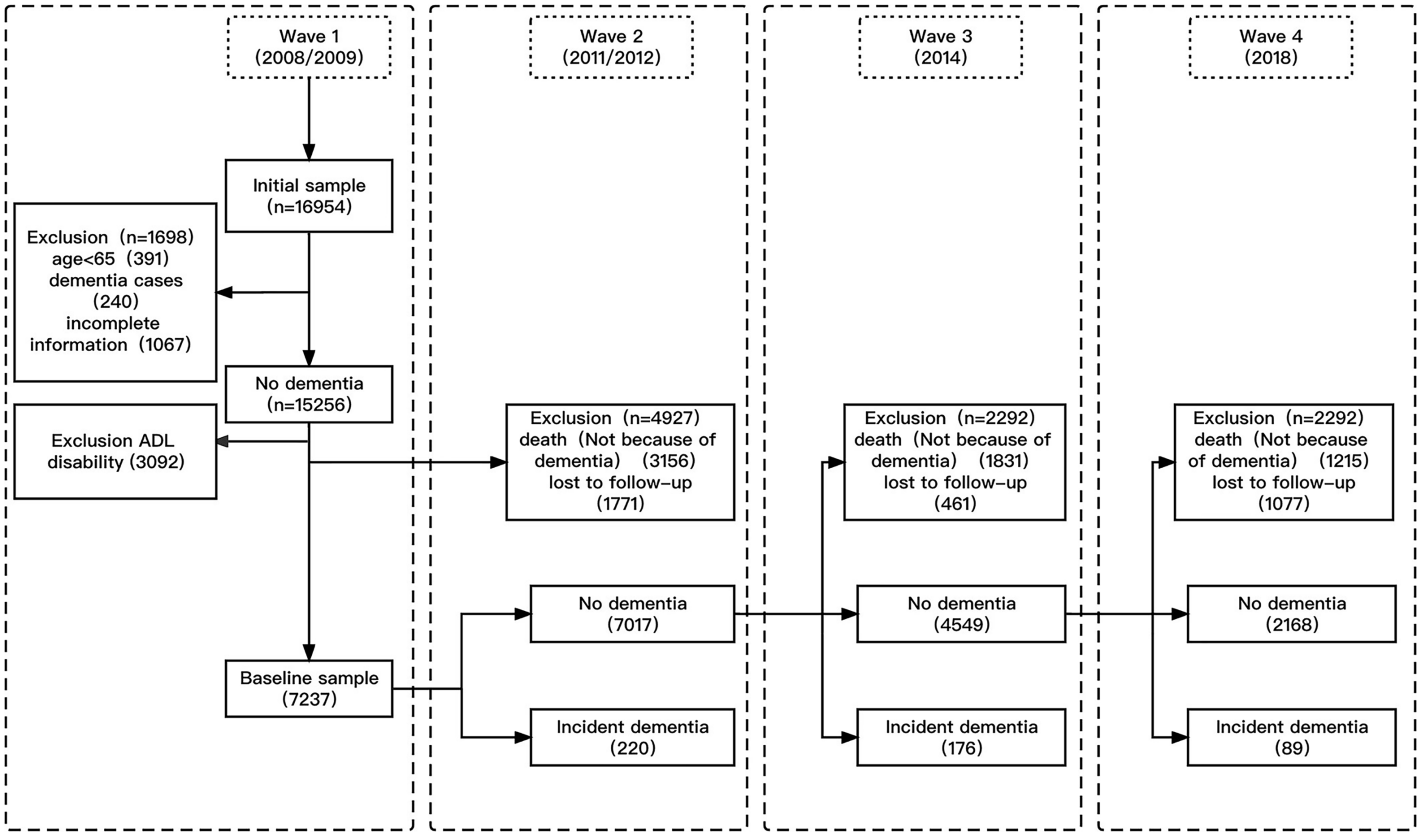
Flow chart of the study population.

The present study was conducted with data from CLHLS participants aged ≥ 65 years with available lifestyle and health status data from 2008, four follow-up time points were conducted in 2008/2009, 2011/2012, 2014, and 2018, with a total follow-up time of up to 10 years. In wave 1 of the CLHLS (2008/2009), the total sample consisted of 16,954 participants. Data from 391 participants aged < 65 years, 240 participants with dementia, and 1,067 participants with incomplete baseline information were excluded from the present study. As participants with ADL disabilities had lost basic self-care abilities, they were also excluded from our study (*n* = 3,092). In addition, individuals lost to follow up (*n* = 1771) and those who died of causes unrelated to dementia (*n* = 3,156) before wave 2 (2011/2012) were excluded from this study. Case shedding is completely random in records and data storage. The cause of shedding is not related to the clinical analysis itself. Therefore, no fill with missing values, missing records generated by the shedding cases are directly discarded to form a complete data set. The final sample for this study thus consisted of 7,237 wave-1 and -2 participants who were additionally evaluated in 2014 and 2018. The sample screening process is illustrated in Figure 1.

### Assessment of housework frequency

At baseline, CLHLS participants were asked “do you do housework at present?” and rated the frequency of doing housework as “almost every day,” “not daily, but once a week,” “not weekly, but at least once a month,” “not monthly, but sometimes,” and “never.” The latter two categories were combined into a “rarely or never” category.

### Identification of dementia

Dementia identified were based on self-reported or proxy-reported physicians’ diagnoses, including, ‘Are you suffering from dementia?’ and ‘Do you have a medical record of dementia diagnosed by a physician?’ Only participants who responded “yes” to both questions were considered to have dementia, previous studies have been validated that the diagnosis of dementia can ensure the requirements of a cohort study (Zhou et al., 2018; Sun et al., 2022; Tian et al., 2022). For those who died during the follow-up but had dementia before death, the reliability of the data was reported by the family and qualified medical institution.

### Evaluation of other variables

To control for potential confounding, we conducted the analysis with covariates representing participants’ demographic, socioeconomic characteristics, lifestyle and health factors, and other types of PA. Specifically, these were age (65–74, 75–84, 85–94, and > 95 years), sex (male/female), marital status (married/not married), education level (no formal education, 1–5 years, and ≥ 6 years), living arrangement (not alone/alone), residential setting (urban/rural), economic level (rich, usual, and poor), body mass index (< 18.5, 18.5–23.9, 24–27.9, and > 28 kg/m2; Pan et al., 2021), sleep duration (< 6, 6–9, and > 9 h), regular exercise (no/yes), alcohol consumption (no/yes), smoking habit (no/yes), hypertension (no/yes), diabetes (no/yes), heart disease (no/yes), stroke or CVD (no/yes), garden work (no/yes), playing card/mahjong (no/yes), and community activity (no/yes).

### Statistical analysis

When describing the baseline characteristics of the baseline sample, the chi-squared test was used to compare categorical variables according to the different groups of housework frequency and with or without dementia classification variable count and percentage. A crude dementia incidence rate was obtained by dividing the number of dementia cases by the total follow-up time, calculated as 1,000 person-years (years from baseline until dementia identification or the last follow-up with no dementia). To evaluate the relationship between doing housework and the risk of dementia, Cox proportional-hazards models were used, with the calculation of hazard ratios (HRs) and 95% confidence intervals (CIs). Model 1 was unadjusted, model 2 was adjusted for demographic characteristics, model 3 was further adjusted for socioeconomic characteristics, and model 4 was further adjusted for lifestyle and health conditions and other types of PA.

We conducted subgroup analyses to explore the effects of gender, age, and regular exercise on the relationship between the frequency of housework and the risk of dementia. To ensure the dependability and stability of the results and exclude redundant interference, we performed a sensitivity analysis. We first excluded participants with epilepsy or stroke at baseline, and then those who developed dementia in the first year. The data preprocessing and statistical analysis were carried out using SPSS (version 24.0) and R (version 4.0.5) with the tidy verse and epi extension packages. Two-sided *p* values < 0.05 were considered to be significant.

## Results

### Participant characteristics

Table 1 shows the participants’ baseline characteristics. The mean age was 81.88 (standard deviation, 10.45) years and 3,824 (52.8%) participants were women. About one-third (32.1%) of participants did housework “rarely or never”; 3.0% did housework “sometimes,” 8.5% did housework “regularly,” and 56.3% did housework “almost every day.” Compared with other groups, more participants who rarely or never did housework were aged > 95 years (23.0%), were married (65.1%), slept ≥ 10 h/night (29.2%), had stroke or CVD (5.3%), and did not exercise regularly (31.6%). The residence setting and incidences of hypertension (*p* = 0.538), diabetes (*p* = 0.542), and heart disease (*p* = 0.899) did not differ among groups (Table 1). Over the follow-up period, 485 participants were diagnosed with dementia. Compared with those without dementia, more participants with dementia were married and living together (68.9% vs. 56.0%), were aged > 85 years (61.6% vs. 39.6%), were women (58.6% vs. 52.4%), slept ≥ 10 h/night (27.8% vs. 22.5%), had no education (63.5% vs. 54.9%), and did not exercise regularly (29.5% vs. 35.0%).

**Table 1:** Demographic characteristics and dementia status at end of follow-up at baseline (*N* = 7,237).

Variable	Frequency of doing housework					Dementia status at end of follow-up		
	Rarely or never%	Sometimes, at least once a month%	Regularly, at least once a week%	Almost everyday%	*p* value	No dementia	dementia	*p* value
Participant, *N*	2,322	220	618	4,077		6,752	485	
Age, *N* (%)					<0.001			<0.001
65~	345 (14.9)	60 (27.3)	186 (30.1)	1,590 (39.0)		2,130 (31.5)	51 (10.5)	
75~	503 (21.7)	68 (30.9)	189 (30.6)	1,326 (32.5)		1951 (28.9)	135 (27.8)	
85~	941 (40.5)	70 (31.8)	171 (27.7)	919 (22.5)		1890 (28.0)	211 (43.5)	
95~	533 (23.0)	22 (10.0)	72 (11.7)	242 (5.9)		781 (11.6)	88 (18.1)	
Sex (male), *N* (%)	1,261 (54.3)	145 (65.9)	384 (62.1)	1,623 (39.8)	<0.001	3,212 (47.6)	201 (41.4)	0.009
Married, *N* (%)	1,512 (65.1)	114 (51.8)	298 (48.2)	2,188 (53.7)	<0.001	3,778 (56.0)	334 (68.9)	<0.001
Education, *N* (%)					0.002			<0.001
NO formal	1,327 (57.1)	109 (49.5)	299 (48.4)	2,280 (55.9)		3,707 (54.9)	308 (63.5)	
1 ~ 5 years	570 (24.5)	62 (28.2)	169 (27.3)	1,031 (25.3)		1718 (25.4)	114 (23.5)	
Above 6 years	425 (18.3)	49 (22.3)	150 (24.3)	766 (18.8)		1,327 (19.7)	63 (13.0)	
Living (alone), *N* (%)	175 (7.5)	25 (11.4)	31 (5.0)	1,028 (25.2)	<0.001	1,170 (17.3)	89 (18.4)	0.566
Economic, *N* (%)					<0.001			0.008
Rich	388 (16.7)	43 (19.5)	92 (14.9)	478 (11.7)		955 (14.1)	46 (9.5)	
Usual	1,601 (68.9)	150 (68.2)	453 (73.3)	2,882 (69.2)		4,663 (69.1)	363 (74.8)	
Poor	333 (14.3)	27 (12.3)	73 (11.8)	777 (19.1)		1,134 (16.8)	76 (15.7)	
Residence (urban), *N* (%)	916 (39.4)	80 (36.4)	221 (35.8)	1,511 (37.1)	0.18	2,546 (37.7)	182 (37.5)	0.936
BMI, *N* (%)					<0.001			0.274
< 18.5	722 (31.1)	52 (23.6)	158 (25.6)	1,037 (25.4)		1819 (26.9)	150 (30.9)	
18.5~	1,251 (53.9)	134 (60.9)	354 (57.3)	2,337 (57.3)		3,813 (56.5)	263 (54.2)	
24~	291 (12.5)	28 (12.7)	87 (14.1)	574 (14.1)		921 (13.6)	59 (12.2)	
28~	58 (2.5)	6 (2.7)	19 (3.1)	129 (3.2)		199 (2.9)	13 (2.7)	
Sleep, *N* (%)					<0.001			0.014
< 6 h	249 (10.7)	20 (9.1)	65 (10.5)	545 (13.4)		816 (12.1)	63 (13.0)	
6–9 h	1,395 (60.1)	161 (73.2)	408 (66.0)	2,738 (67.2)		4,415 (65.4)	287 (59.2)	
> 9 h	678 (29.2)	39 (17.7)	145 (23.5)	794 (19.5)		1,521 (22.5)	135 (27.8)	
Current smoker, *N* (%)	517 (22.3)	51 (23.2)	168 (27.2)	816 (20.0)	<0.001	1,459 (21.6)	93 (19.2)	0.207
Current drinker, *N* (%)	501 (21.6)	45 (20.5)	146 (23.6)	793 (19.5)	0.044	1,392 (20.6)	93 (19.2)	0.448
Regular exercise, *N* (%)	733 (31.6)	78 (35.5)	260 (42.1)	1,432 (35.1)	<0.001	2,360 (35.0)	143 (29.5)	0.014
Hypertension, *N* (%)	487 (21.0)	45 (20.5)	120 (19.4)	889 (21.8)	0.538	1,429 (21.2)	112 (23.1)	0.316
Diabetes, *N* (%)	54 (2.3)	4 (1.8)	16 (2.6)	116 (2.8)	0.542	179 (2.7)	11 (2.3)	0.61
Heart disease, *N* (%)	204 (8.8)	20 (9.1)	49 (7.9)	360 (8.8)	0.899	593 (8.8)	40 (8.2)	0.687
Stroke or CVD, *N* (%)	123 (5.3)	8 (3.6)	30 (4.9)	166 (4.1)	0.028	298 (4.4)	29 (6.0)	0.109
Garden work, *N* (%)	161 (6.9)	20 (9.1)	58 (9.4)	540 (13.2)	<0.001	750 (11.1)	29 (6.0)	<0.001
Playing card/mahjong, *N* (%)	165 (7.1)	16 (7.3)	41 (6.6)	325 (8.0)	0.483	529 (7.8)	18 (3.7)	0.001
Community activity, *N* (%)	52 (2.2)	3 (1.4)	21 (3.4)	166 (4.1)	<0.001	231 (3.4)	11 (2.3)	0.172

Missing data were not shown in this table. Missing data were not analyzed. N, number; BMI, body mass index; CVD, cerebrovascular disease.

### Association of housework with the risk of dementia

The dementia incidence rate per 1,000 person-years decreased gradually as the frequency of housework increased (Table 2). This rate was 13.7 (95% CI, 12.0–15.4) among participants who rarely or never did housework, 9.3 (95% CI, 5.4–14.8) for those who did housework at least once a month, 7.4 (95% CI, 5.3–10.2) for those who did housework at least once a week, and 5.1 (95% CI, 4.4–5.9) for those who did housework almost every day. The unadjusted HR for dementia was significantly lower for participants who did housework almost daily than for those who rarely or never did housework (0.39; 95% CI, 0.32–0.47). This association was attenuated after adjustment in models 2 (0.50; 95% CI, 0.41–0.62), 3 (0.48; 95% CI, 0.38–0.59), and 4 (0.49; 95% CI, 0.39–0.61). These results suggest that the regular performance of housework has a strong protective effect against dementia among older Chinese adults.

**Table 2:** Associations between frequency of doing housework and dementia for Chinese older adults.

Frequency of doing housework	*N* cases/*N* total	ID (95%CI) per 1,000 persons-years	Model 1 HR (95% CI)	*p* value	Model 2 HR (95% CI)	*p* value	Model 3 HR (95% CI)	*p* value	Model 4 HR (95% CI)	*p* value
Rarely or never	246/2322	13.7 (12.0–15.4)	Ref		Ref		Ref		Ref	
Sometimes^a^	17/220	9.3 (5.4–14.8)	0.70 (0.43–1.14)	0.147	0.83 (0.50–1.35)	0.446	0.83 (0.51–1.37)	0.469	0.86 (0.52–1.41)	0.549
Regularly^b^	39/618	7.4 (5.3–10.2)	0.56 (0.40–0.79)	0.001	0.68 (0.48–0.95)	0.026	0.68 (0.48–0.95)	0.024	0.69 (0.49–0.98)	0.036
almost everyday	183/4077	5.1 (4.4–5.9)	0.39 (0.32–0.47)	<0.001	0.50 (0.41–0.62)	<0.001	0.48 (0.38–0.59)	<0.001	0.49 (0.39–0.61)	<0.001

a Sometimes at least once for a month.

b Regularly at least once for a week.

*N*, number; Ref, reference; ID, Incidence density; HR, hazard ratio; CI, Confidence interval.

Model 1 is unadjusted.

Model 2 adjusted for age and sex.

Model 3 adjusted for age, sex, marital status, education, living arrangement and economic level.

Model 4 adjusted for age, sex, marital status, education, living arrangement, economic level, BMI, sleeping duration, smoking status, drinking status, exercise, Stroke or cerebrovascular disease, garden work, playing card/mahjong, community activity.

### Subgroup findings

**Figure 2:**
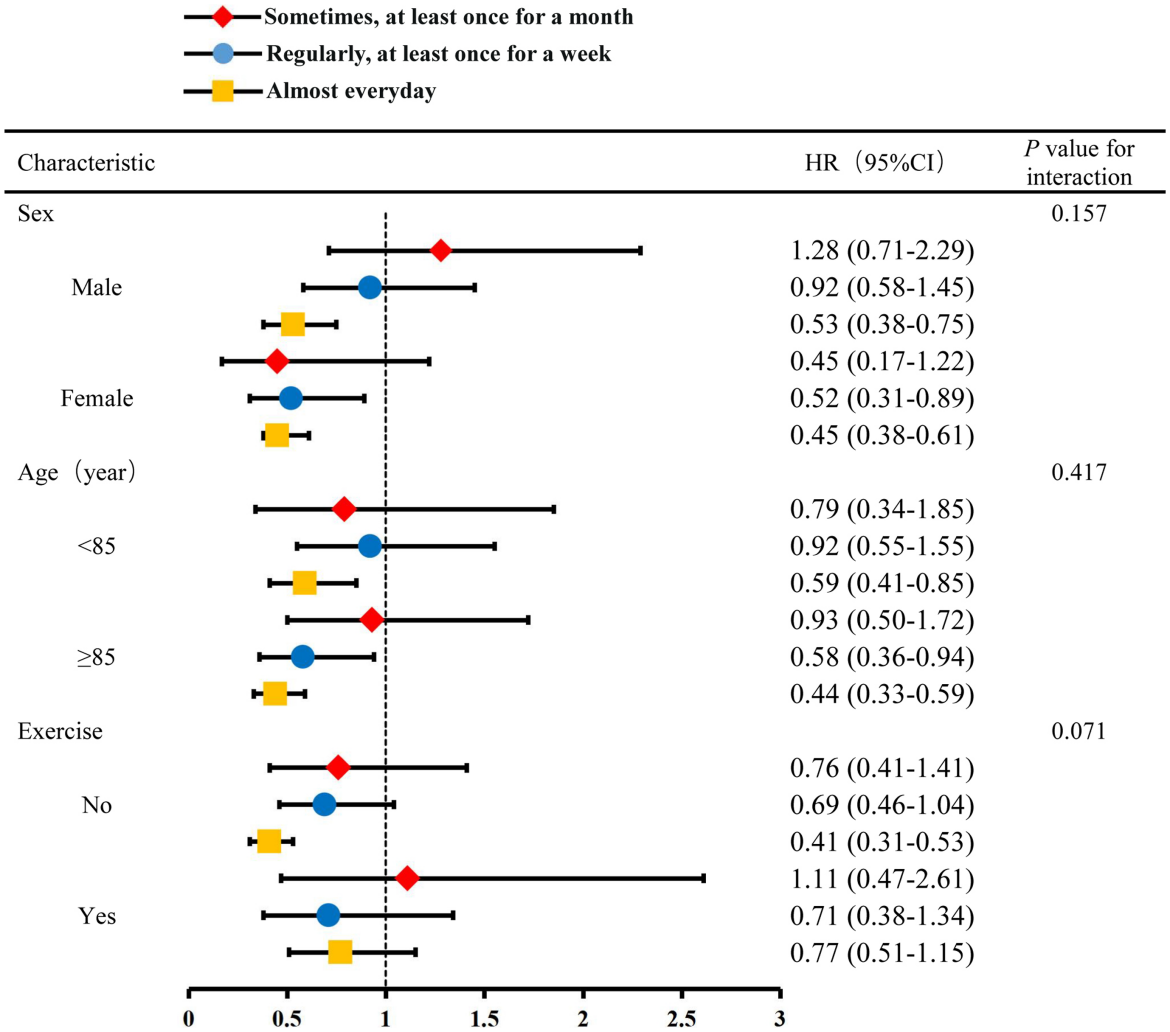
Hazard ratios (HRs) and confidence intervals (CIs) for the association between frequency of doing housework and dementia, with rarely or never doing housework as a reference group, by baseline, age, sex, and exercise, adjusting for baseline age, sex, marital, education, living arrangement, economic level, BMI, sleeping duration, smoking status, drinking status, exercise, Stroke or cerebrovascular disease, garden work, playing card/mahjong, community activity.

The performance of housework almost every day significantly reduced the risk of dementia for males (HR = 0.53; 95% CI, 0.38–0.75) and females (HR = 0.45; 95% CI, 0.38–0.61) and for those aged < 85 years (HR = 0.59; 95% CI, 0.41–0.85) and ≥ 85 years (HR = 0.44; 95% CI, 0.33–0.59; Figure 2). In addition, frequently doing housework reduced the incidence of dementia among participants who did not exercise regularly (HR = 0.41; 95% CI, 0.31–0.53), there is no evidence shown an interaction between whether the elderly Housework and sex, age, regular exercise.

## Discussion

This study is the first to identify an association between the frequency of housework activities and the dementia risk among older Chinese adults, filling a research gap. We selected a high-level and previously validated CLHLS database (Gu et al., 2009; Gu and Feng, 2015), showed that a higher frequency of housework reduced the dementia risk, and that this effect was more significant among people who did not exercise regularly than among those who did otherwise. The results of subgroup analyses stratified according to age and sex as well as sensitivity analyses did not differ substantially from those of the main analyses.

Our results generally agreed with those of previous studies. A cross-sectional survey of community-dwelling older adults in Singapore showed that the intensity of housework was related to higher cognitive levels, especially regarding attention and memory, and that housework was related to physical functioning and sensorimotor performance (Lee et al., 2021). In that study, the Battery for the Assessment of Neuropsychological Status (BRANS) scores of individuals with vigorous and moderate housework activity levels were 8 and 5% higher, respectively, than those of individuals with low housework activity levels (Lee et al., 2021). Similarly, in a United Kingdom Biobank cohort of > 500,000 people followed for > 10 years, the risk of developing various types of dementia was 21% lower among people who engaged in moderate-to high-intensity housework activities than for those who performed low-intensity housework (Zhu et al., 2022). This protective effect was less pronounced than that observed in the East Asian population examined in the present study, perhaps due to ethnic and cultural differences. However, other studies had different results. A review of 30 studies revealed that evidence was insufficient to argue that PA or exercise improves the cognitive ability of older adults (Snowden et al., 2011). Potential contributors to these inconsistent results include heterogeneous PA classification and measurement, examination of small samples, short follow-up periods, and lack of stratified analysis focusing on regular exercise. In this study, we applied detailed data from the CLHLS that covered sociodemographic, lifestyle, and health factors, as well as other types of PA with > 10 years of follow-up for a large sample of older adults, which enabled stratified analysis based on age and gender, gender difference may have a bearing on sex-specific differences in amygdala and hippocampal volume and The sex hormone-dependent of brain regional development (Neufang et al., 2009; Giedd et al., 2012).

The UN General Assembly declared 2021–2030 the Decade of Healthy Ageing, highlighting the importance for prevent aging and disease (Chen et al., 2022). Sufficient evidence demonstrates the mental and physical health benefits of recreational PA (Thacker et al., 2008; White et al., 2016), in the current era and environmental context, people prefer to prevent aging and disease by having a healthy lifestyle and participating in moderate exercise instead of relying on drugs (De la Rosa et al., 2020; Fang et al., 2020), However, the effect of non-recreational PA on the incidence of dementia still requires additional research. Caregiver or professional guidance is required for patients with dementia to engage in most PA. Housework is a common activity of daily living (ADL) that does not require strict oversight, making it a more convenient and less costly way for these patients to engage in PA.

Stroke is considered a risk factor for dementia (Rost et al., 2022), we first excluded participants with stroke or CVD at baseline. To minimize the possibility of reverse causality in the observed associations, we then excluded participants who developed dementia during the first year of follow-up. The results of the sensitivity analyses were similar to those of the main analyses (Table 3). Housework was the sole factor examined in this study, which involved innovative classification as well as quantitative evaluation and adjustment for potential confounding factors. This revealed for the first time that a low frequency of housework was associated with an increased dementia risk among older Chinese adults.

**Table 3:** Association of frequency of doing housework with dementia after further exclusions.

Frequency of doing housework	*N* cases/*N* total	ID (95%CI) per 1,000 persons-years	Model 1 HR (95% CI)	*p* value	Model 2 HR (95% CI)	*p* value	Model 3 HR (95% CI)	*p* value	Model 4 HR (95% CI)	*p* value
Excluding participants who suffer from stroke or CVD at baseline										
Rarely or never	258/2724	13.8 (12.0–15.6)	Ref		Ref		Ref		Ref	
Sometimes^a^	19/210	9.0 (5.1–14.6)	0.67 (0.40–1.11)	0.12	0.81 (0.48–1.34)	0.4	0.81 (0.49–1.35)	0.43	0.83 (0.50–1.39)	0.478
Regularly^b^	32/576	7.6 (5.4–10.4)	0.57 (0.40–0.80)	0.001	0.69 (0.49–0.98)	0.04	0.69 (0.49–0.98)	0.04	0.71 (0.50–1.00)	0.055
almost everyday	164/3818	4.9 (4.2–5.7)	0.37 (0.30–0.45)	<0.001	0.48 (0.39–0.60)	<0.001	0.45 (0.36–0.57)	<0.001	0.46 (0.37–0.58)	<0.001
Further excluding dementia that occurred during the first year of follow-up										
Rarely or never	84/2713	13.0 (11.3–14.8)	Ref		Ref		Ref		Ref	
Sometimes^a^	10/209	8.4 (4.7–13.9)	0.67 (0.39–1.12)	0.127	0.80 (0.47–1.35)	0.394	0.80 (0.47–1.36)	0.412	0.83 (0.49–1.40)	0.474
Regularly^b^	20/597	7.6 (5.40–10.5)	0.60 (0.43–0.85)	0.004	0.73 (0.52–1.03)	0.074	0.73 (0.51–1.03)	0.073	0.75 (0.53–1.06)	0.105
almost everyday	87/3929	4.8 (4.1–5.6)	0.38 (0.31–0.47)	<0.001	0.49 (0.40–0.61)	<0.001	0.47 (0.37–0.59)	<0.001	0.48 (0.38–0.60)	<0.001

a Sometimes at least once for a month.

b Regularly at least once for a week.

*N*, number; Ref, reference; ID, Incidence density; HR, hazard ratio; CI, Confidence interval.

Model 1 is unadjusted.

Model 2 adjusted for age and sex.

Model 3 adjusted for age, sex, marital status, education, living arrangement and economic level.

Model 4 adjusted for age, sex, marital status, education, living arrangement, economic level, BMI, sleeping duration, smoking status, drinking status, exercise, Stroke or cerebrovascular disease, garden work, playing card/mahjong, community activity.

This study has several important limitations. First, information about participants’ dementia status and the covariates was obtained by referencing self-reports and questionnaires. Thus, information bias and survey bias may have affected the results. However, due to the support of a sufficiently large sample size, this is within the tolerable range. Second, our findings are applicable only to older Chinese adults. As the effects of housework frequency on the incidence of dementia may differ between younger and older adults, extending the conclusions to the former group should be done with caution. Third, we collected data only on the frequency of housework activities without specifically classifying them. We were unable to characterize the intensity of housework or examine the effects of specific activities on the incidence of dementia. Future research should focus on collecting more specific examples of this information.

## Conclusion

The findings of this study suggest that the frequent performance of housework is associated with a reduced incidence of dementia among adults aged > 65 years in China, suggesting that this habit promotes healthy aging in this population. Interventional and longitudinal studies are needed to explore the relativity of the relationship between housework and the risk of dementia.

## Data availability statement

The datasets presented in this study can be found in online repositories. The names of the repository/repositories and accession number(s) can be found at: https://cpha.duke.edu/research/chinese-longitudinal-healthy-longevity-survey-clhls.

## Ethics statement

The studies involving human participants were reviewed and approved by the datasets used in this study were obtained from the China Longitudinal Healthy Longevity Survey and reviewed by Peking University. The study was approved by the Research Ethics Committee of Peking University (IRB00001052-13074). All study participants provided written informed consent prior to the completion of the investigation. The patients/participants provided their written informed consent to participate in this study.

## Author contributions

YjW and YZ had full access to all of the data used in this study and take responsibility for the integrity of the data and the accuracy of the data analysis. YlW and XLu performed the data analysis and drafted the manuscript. XLo and YS checked the accuracy of the data analysis. SZ was responsible for data entry. All authors take responsibility for all aspects of the article and guarantee the accuracy and completeness of the reporting of findings. All authors contributed to the article and approved the submitted version.

## Funding

This work was supported by Shenzhen Fund for Medical Research Foundation of Guangdong Province, Project number: B2021086 and Guangdong Provincial High-level Clinical Key Specialties (No. SZGSP013) and Guangdong Basic and Applied Basic Research Foundation (no. 2020A1515011469).

## Conflict of interest

The authors declare that the research was conducted in the absence of any commercial or financial relationship that could be construed as a potential conflict of interest.

## Publisher’s note

All claims expressed in this article are solely those of the authors and do not necessarily represent those of their affiliated organizations, or those of the publisher, the editors and the reviewers. Any product that may be evaluated in this article, or claim that may be made by its manufacturer, is not guaranteed or endorsed by the publisher.
